# Meeting the Therapeutic Challenges of Emergent and Rare Invasive Fungal Diseases Through Novel Clinical Trial Designs

**DOI:** 10.1093/ofid/ofae257

**Published:** 2024-06-17

**Authors:** Thomas J Walsh

**Affiliations:** Center for Innovative Therapeutics and Diagnostics, Office of the Director (citdx.org), Richmond, Virginia, USA; Departments of Medicine and of Microbiology & Immunology, University of Maryland School of Medicine, Baltimore, Maryland, USA

**Keywords:** clinical trials, invasive fungal diseases, new antifungal agents, rare diseases, emergent invasive fungal diseases

## Abstract

Treatments for emerging and rare invasive fungal diseases (IFDs) represent a critical unmet medical need. For IFDs that occur less frequently than invasive aspergillosis, such as mucormycosis, hyalohyphomycosis, and phaeohyphomycosis, randomized controlled clinical trials are impractical and unlikely to meet urgent public health needs. Understanding regulatory approaches for approval of drugs for rare cancers and rare metabolic diseases could help meet the challenges of studying drugs for rare IFDs. A single-arm, controlled clinical trial with a high-quality external control(s), with confirmatory evidence from nonclinical studies, including pharmacokinetic/pharmacodynamic data in predictive animal models of the disease may support findings of effectiveness of new drugs and biologics. Control populations may include historical controls from published literature, patient registries, and/or contemporaneous external control groups. Continuous engagement among clinicians, industrial sponsors, and regulatory agencies to develop consensus on trial design and innovative development pathways for emergent and rare invasive fungal diseases is important.

## Teaser

A single, adequate, and well-controlled clinical trial with confirmatory evidence from nonclinical studies, including predictive animal models with supportive pharmacokinetic/pharmacodynamic data, should be considered as evidence of effectiveness of novel treatments for emerging and rare invasive fungal diseases.

A standard noninferiority clinical trial evaluating therapies for the treatment of invasive pulmonary aspergillosis (IPA) currently requires approximately 5 years and approximately 100 institutions to enroll approximately 500 patients [1, 2]. For invasive fungal diseases (IFDs) that occur considerably less frequently than IPA, such as mucormycosis, fusariosis, scedosporiosis, and rare yeast infections, as well as uncommon or refractory endemic mycoses, traditional randomized, controlled, double-blind clinical trials are impractical and unable to rapidly address the urgent public health burden and suffering posed by these emerging infectious diseases.

For regulatory purposes, a rare disease is defined as occurring in <200 000 people in the United States [3]; however, most rare diseases occur in much lower numbers, and the same is true for rare IFDs. Indeed, the rare IFDs discussed herein may be called “ultrarare” diseases [4, 5]. For these rare IFDs, there is perhaps another possible approach toward evaluating the efficacy of antifungal drugs and finding a more feasible development pathway, emulating the process for evaluation of therapeutic interventions in rare cancers as well as rare metabolic diseases. Therefore, a review and assessment of development pathways used for novel therapeutics for rare cancers, as well as those for rare metabolic diseases, is warranted as a potential viable regulatory pathway to meeting the challenges of serious infectious diseases caused by the rare molds and yeasts that are continuing to emerge as global threats to public health.

In addition to development of novel antifungal agents, another approach to meeting the challenges of rare but potentially lethal IFDs is through repurposing drugs that were originally developed for other indications. Among alternative innovative strategies for clinical research is target trial emulation, which has been used in other diseases.

These uncommon but emerging fungal pathogens include yeasts (eg, *Trichosporon, Rhodotorula, Exophiala, Magnusiomyces,* and *Saprochaete* species), relatively new *Candida* species (eg, *Candida auris* and *Candida haemulonii*), Mucormycetes (eg, *Rhizopus, Rhizomucor, Mucor, Lichtheimia, Apophysomyces, Saksenaea, and Cunninghamella* species), hyaline molds (eg, *Fusarium, Paecilomyces,* and *Purpureocillium* species), dematiaceous molds (eg, *Scedosporium, Lomentospora, Cladophialophora*, *Exserohilum*, and *Rhinocladiella* species), and some emerging thermally dimorphic molds (eg, *Emergomyces* species) [6, 7].

Susceptible hosts include immunocompromised patients with neutropenia and or T-cell dysfunction related to hematologic cancers, hematopoietic cell transplantation (HCT), solid organ transplantation, and human immunodeficiency virus (HIV) or AIDS. There is also an expanding host population recognized as vulnerable to mucormycosis and other rare molds, such as patients with diabetes mellitus or severe coronavirus disease 2019 [8]. Some rare fungal pathogens cause disease in otherwise healthy patients with trauma [9]. Among non-*Aspergillus* molds, Mucormycetes followed by *Fusarium*, *Lomentospora, and Scedosporium* are the most common filamentous fungi causing IFDs among patients with hematopoietic cell transplant (HCT) and solid organ transplants in the United States [10].

The incidence and prevalence of rare IFDs varies depending on the underlying illness, the institution, and geographic region. The prevalence of human mucormycosis is estimated at 2 cases per million in the United States, with other non-*Aspergillus* molds being less common [11]. Prospective surveillance among 1063 solid organ transplant recipients with 1208 IFDs conducted in 23 institutions in the United States during 2001–2006 found that mucormycosis accounted for only 2% of all IFDs [12]. By comparison, prospective surveillance among 875 HCT recipients with 983 IFDs in the United States found that mucormycosis accounted for 8% of all IFDs in this patient population [13].

Other rare but emerging invasive mold diseases also present serious threats to the expanding population of immunocompromised patients. Sinopulmonary and disseminated disease caused by *Fusarium* spp occurred in 6 per 1000 HCT recipients (0.6%) overall and in up to 20 per 1000 (2%) of allogeneic HCT recipients with HLA-mismatched related donor, in Brazil and the United States, respectively [14]. In one US multicenter study, fusariosis occurred in 2 (4%) of 53 recipients of liver and cardiac transplants with invasive mold diseases [15]. *Lomentospora prolificans* and *Scedosporium apiospermum* infections were reported in 2% and 6%, respectively, of the recipients of liver and cardiac transplants in the same US study [15]. *L prolificans* disease accounted for 8 of 23 non-*Aspergillus* mold infections (35%), over a 3-year period, in patients with varying underlying diseases in one US health system [16]. While another US single-center study of *Scedosporium*/*Lomentospora* disease in a cohort of patients with hematologic cancer or HCT reported 25 proven or probable cases (1989–2006) with an incidence of 1.11 cases per 100 000 patient-inpatient days and increasing to 1.33 per 100 000 patient-inpatient days (1999–2005) [17].

Life-threatening IFDs caused by resistant yeast infections are also increasing. *C auris* infections were first reported in the United States in 2016 and became a notifiable disease in 2018 [18, 19]. As of 31 December 2021, a total of 3270 clinical cases in the United States were reported to the Centers for Disease Control and Prevention [20]. The number of reported clinical cases increased by 44% in 2019 and by 95% in 2021. Seventeen states reported their first cases of *C auris* during this interval. In parallel with this increased incidence of *C auris* infections, the frequency of echinocandin resistance increased approximately 3-fold [20]. With increasing echinocandin resistance, panfungal resistant strains of *C auris* are potentially lethal pathogens [21]. With the expanding use of echinocandins for antifungal prophylaxis in patients with hematologic cancers, serious infections caused by echinocandin-resistant *Candida* spp and *Trichosporon asahii* also are emerging.

## DRUG APPROVALS FOR RARE IFDS

The limited treatment options for rare IFDs represent an area of critical unmet medical need. Published global guidelines on the clinical management of rare invasive yeasts and mold infections highlighted these limited treatment options, knowledge gaps, and constraints in management that require further investigation [22–24].

In the past 60 years, 4 drugs: amphotericin B deoxycholate, amphotericin B lipid complex, voriconazole, and isavuconazonium sulfate, were approved, depending on the antifungal agent, by the US Food and Drug Administration (FDA) for the treatment of mucormycosis and other rare IFDs (Table 1) [25–29]. While the basis of approval of antifungal drugs for invasive candidiasis and IPA has evolved from open-label trials to randomized, controlled trials, the approval of treatments for less common IFDs has been based on noncomparative, open-label studies or case series depending on the drug. Isavuconazonium sulfate was approved in 2015 for the primary treatment of invasive aspergillosis and mucormycosis. The approval for mucormycosis was supported by a randomized, controlled trial of isavuconazonium compared with voriconazole in 516 patients with invasive aspergillosis and an open-label, noncomparative trial that evaluated the safety and efficacy of the drug in 37 patients with invasive mucormycosis [1, 29].

**Table 1.** Drugs Approved by the US Food and Drug Administration for Rare Invasive Fungal Diseases, 1959–2015

Antifungal Drug; Year of Approval	Basis for FDA Approval	Indication(s)	Trial: No. of Participants	Primary End Point
Amphotericin B [24]; 1959	Open-label, noncomparative data	Treatment of potentially life-threatening fungal infections: aspergillosis, cryptococcosis (torulosis), North American blastomycosis, systemic candidiasis, coccidioidomycosis, histoplasmosis, zygomycosis, including mucormycosis due to susceptible species of the genera *Absidia*, *Mucor,* and *Rhizopus*, and infections due to related susceptible species of *Conidiobolus* and *Basidiobolus* and sporotrichosis; amphotericin B may be useful for treating American mucocutaneous leishmaniasis, but it is not the drug of choice as primary therapy	Unavailable	Unavailable
Amphotericin B lipid complex (Abelcet) [25]; 1995	Efficacy and safety data from 473 patients (282 evaluable) in 3 open-label studies	Treatment of invasive fungal infections in patients refractory to or intolerant of conventional amphotericin B therapy	Invasive fungal infections: 473 (282 evaluable)	Global response criteria
Voriconazole (Vfend) [26]; 2002	Phase 3 randomized, controlled clinical trial of voriconazole vs amphotericin B in 277 patients with IA and noncomparative study of 24 patients with *Scedosporium* and 21 with *Fusarium* infections	Treatment of IA, candidemia, and other deep-tissue *Candida* infections, esophageal candidiasis, fusariosis, and scedosporiosis	IA trial: 277 Rare molds: 45	Global response criteria
Isavuconazonium sulfate (Cresemba) [27]; 2015	Randomized, controlled trial of isavuconazonium vs voriconazole in 516 patients with IA and open-label, noncomparative trial evaluating the drug’s safety and efficacy in 37 patients with invasive mucormycosis	Treatment of IA and invasive mucormycosis	IA trial: 516; invasive mucormycosis: 37	All-cause mortality rate

Abbreviation: IA, invasive aspergillosis.

For the single-arm, open-label study of mucormycosis, natural history data were used as a basis of comparison to assess efficacy. The new drug application contained a comparative analysis of matched, contemporaneously treated controls from an established fungal registry as supportive evidence of efficacy and safety. The all-cause mortality rate through day 42 (primary end point) in patients with mucormycosis was 38%, and the overall clinical response at the end of treatment was 31%. These results provided sufficient evidence that isavuconazonium was effective for the treatment of mucormycosis, considering the natural history of untreated mucormycosis reported in published literature [30, 31]. Data on the activity of isavuconazonium in murine models of pulmonary mucormycosis were also supportive of its efficacy. The activity of isavuconazonium was measured in neutropenic and diabetic ketoacidotic Institute for Cancer Research mice infected by the inhalational (pulmonary infection model of mucormycosis) or intravenous (hematogenous dissemination infection model of mucormycosis) routes with a strain of *Rhizopus oryzae* (*Rhizopus delemar*). In the pulmonary infection model of mucormycosis, isavuconazonium was as effective as high-dose liposomal amphotericin B in protecting infected neutropenic mice but not diabetic ketoacidotic mice [29, 32]. The active-controlled clinical trial in invasive aspergillosis provided the preponderance of safety data for isavuconazonium.

Recently developed novel antifungal agents have been found to have in vitro and in vivo antifungal activity against rare molds, including the Mucormycetes, *L prolificans*, *Scedosporium* spp, *Fusarium* spp, triazole-resistant *Aspergillus* spp, and *Scopulariopsis* spp, as well as uncommon yeasts, including *C auris*, *C haemulonii*, *T asahii*, *Magnusiomyces* spp, and *Saprochaete* spp, and endemic dimorphic fungi, such as *Coccidioides* spp and *Emergomyces* spp. These novel antifungal agents that are being studied in clinical trials include but are not limited to encochleated amphotericin B, F901318, fosmanogepix, ibrexafungerp, olorofim, opelconazole, oteseconazole, PC945, rezafungin, SCY-247, and VT-1598 [33–36]. The lessons learned from the approval process of drugs for treatment of rare cancers and inborn errors of metabolism may be helpful for development of novel antifungal agents for management of IFDs caused by less common but life-threatening fungal pathogens.

## DRUG APPROVALS FOR RARE CANCERS AND INBORN ERRORS OF METABOLISM

Understanding regulatory approaches for approval of drugs for rare cancers and rare metabolic diseases could help meet the challenges of studying drugs for rare IFDs. In contrast to the relatively small number of approved drugs for rare IFDs, considerably more drugs have been approved for treatment of a variety of rare cancers and inborn errors of metabolism [37, 38]. Between 1987 and 2011, the FDA approved 45 drugs for 68 rare cancers; only one-third of the 99 trials that supported the drug approvals were randomized, and 63% were based on a single trial with a median sample size of 54 patients [39]. Overall objective response rate (a direct measure of treatment that includes complete and partial response) was the primary efficacy end point in 69% of the approvals, and less frequently used end points included time to progression, progression-free survival, and overall survival. Time-to-events end points such as progression-free survival and overall survival may be difficult to interpret in a single-arm study.

The approvals of some oncologic drugs (2019–2021) for treatment of rare cancers were based on nonrandomized studies. Among 11 selected oncologic drugs approved for the treatment of rare cancers between 2019 and 2021, 3 drugs [40] (belzutifan, avapritinib, and capmatinib) received traditional approval, and 8 drugs [41] (axicabtagene ciloleucel, infigratinib, brexucabtagene autoleucel, selpercatinib, pomalidomide, pralsetinib, entrectinib, and zanubrutinib) received an accelerated approval based on an intermediate clinical end point(s) with supporting evidence from duration of response. There were 1–3 clinical trials per new drug application or biologics license application, with the majority containing 1 single-arm, open-label, multicenter trial. Study enrollments varied in size from 28 to 314 participants. The most common primary end point in these trials was the objective response rate, defined as the achievement of either a partial response or complete response. Efficacy outcomes were assessed by an independent review committee. Accelerated approval requires a surrogate end point that is reasonably likely to predict clinical benefit or an intermediate clinical end point that is a measure of a therapeutic effect. This therapeutic effect may measure the rate of of irreversible disease or death [42]. For drugs approved under this pathway, a clinical study (or studies) to confirm the clinical benefit is (are) a postapproval requirement.

The approvals of selected drugs for treatment of rare inborn errors of metabolism, such as uridine triacetate [43], cerliponase alfa [44], lonafarnib [45, 46], and cyclic pyranopterin monophosphate [47, 48], were also based on single-arm trials of the investigational product, as compared wiith data from matched historical control registries, as well as confirmatory evidence of functional and/or survival outcomes in 2 animal models of the diseases, strong mechanistic evidence, or historical data regarding disease manifestations and pharmacodynamic effects on and off treatment.

## APPROACHES TO THE ASSESSMENT OF EFFICACY OF ANTIFUNGAL AGENTS FOR RARE IFDS

Approval of a drug for any disease must be based on substantial evidence of the drug's effectiveness for its intended use and sufficient information to conclude that the drug is safe for use under the conditions outlined in the proposed labeling [49]. The FDA has approved new drugs for some rare cancers and inborn errors of metabolism based on the results of single controlled trials with confirmatory evidence from other sources such as preclinical studies. Perhaps the strategies used to study rare cancers and inborn errors of metabolism could also be applied to the design of studies for rare IFDs.

Among several possible approaches to the assessment of efficacy of antifungal drugs for rare IFDs would be a controlled clinical trial with confirmatory evidence from nonclinical sources, including predictive animal models of the fungal disease, to demonstrate effectiveness (Table 2). The FDA guidances for industry are useful resources for the conduct of efficient drug development programs [49, 50] and for potential approaches to designing clinical studies for an antifungal drug as primary treatment for rare IFDs.

**Table 2.** Potential Approaches to Evaluation of an Antifungal Drug for Treatment of Rare Invasive Fungal Diseases^a^

Prospective single-arm trial of IFD cases with a control group with IFD from literature
OR
Prospective single-arm trial of IFD cases with matched control group from fungal registry database
OR
Prospective single-arm trial of IFD cases with a matched control group from medical chart review
OR
Prospective 2-arm trial of IFD cases comparing 2 different doses of test drug
OR
Prospective 2-arm trial of IFD cases with the test drug plus standard-of-care drug vs standard-of-care drug plus placebo
OR
Randomized controlled trial with randomization of, eg, 2:1 or 3:1 of study drug vs best available therapy and augmentation of randomized concurrent control group with external control(s)
AND
Study (placebo-controlled) of the test drug in ≥1 animal models of the IFD as supportive evidence of efficacy
AND
Patient-level safety data from phase 1, 2, and 3 clinical studies, fungal registry databases, case series, and case reports

Abbreviation: IFD, invasive fungal disease.

^a^Refer to the Efficacy End Points section for more detailed discussion.

The Limited Population Pathway for Antibacterial and Antifungal Drugs provides the FDA and stakeholders with a tool to help with the approval of antifungal drugs for treatment of serious and life-threatening infections in limited populations of patients with unmet needs such as patients with IFDs [51]. Critical to the successful implementation of this pathway for new antifungal agents is the availability of robust control datasets.

## STUDY CONTROLS

A key element in the assessment of efficacy is an appropriate control population [52]. Moreover, having a reasonable understanding of the natural history of the fungal disease under study is also important. Controls may include a historical control from published literature, patient registry, and/or a contemporaneous external control group. The paucity of high-quality external controls has been a limiting factor in the conduct of clinical trials for the less common IFDs. It may also be possible, concurrent with a clinical trial, to prospectively gather data reflecting standard of care in a group of medical centers for the purposes of understanding contemporary standards of care and for accrual of study controls. Such data could also be collected retrospectively, albeit less ideally. Examples of prospectively collected registries of rare and uncommon diseases include those for rare cancers [53, 54], cystic fibrosis [55], rare genetic diseases [56], inborn errors of metabolism [57], and primary immunodeficiencies [58].

There are few actively enrolling, prospectively collected, registries for IFDs. Among these is the FungiScope registry [59], based at University of Cologne, Cologne, Germany, which played an important role in providing case controls for the evaluation of isavuconazonium for primary treatment of mucormycosis. Other actively enrolling registries include the European Confederation for Medical Mycology–International Society for Human and Animal Mycology Zygomyco.net [60] and the registry of the European Confederation of Medical Mycology Working Group on Zygomycosis (2005 and 2007) [61]. While previous studies of invasive mycoses also enrolled patients through registries, such as TRANSNET [12, 13], PATH Alliance [62–66], and the Mycoses Study Group Education & Research Consortium phaeohyphomycosis project [67], these studies and other registry-based studies [68, 69] are now closed. However, their databases may still be applicable in identifying case controls. Expanding the number of registries with sustained support to prospectively develop databases for rare mold diseases would greatly strengthen the analysis of efficacy of investigational antifungal agents.

As a starting point for study protocol development, the source(s) of the external control should be identified and evaluated for accessible clinical data and for the feasibility of its use for establishing a reasonably well-matched control group before enrollment begins in the planned trial. Ideally, patient-level data should be available for the control population. Defined criteria are necessary to understand which patients will be enrolled in the control group. As many patients will have received prior therapy, matching or adjusting for the following key parameters is important: prior antifungal treatment, extent of IFD (e.g., single organ vs disseminated disease), and other prognostic factors, such as the type of organ transplant, net state of immunosuppression, use of corticosteroids or other immunomodulatory drugs, and status of remission of the underlying neoplastic process in patients with hematologic cancers. Interpretability may be limited if overly restrictive exclusion criteria in the study select for a patient population with fewer measured comorbid conditions than those of historical controls.

More than a single source of controls may be helpful for an assessment of efficacy in a small trial, as was the case for the antituberculosis drug, pretomanid [70]. Pretomanid was approved by the US FDA in 2019 as part of a 3-drug regimen (pretomanid, bedaquiline, and linezolid) for the treatment of two adult populations: (1) those with pulmonary tuberculosis resistant to isoniazid, rifamycins, a fluoroquinolone, and a second-line injectable antibacterial drug and (2) those with pulmonary tuberculosis resistant to isoniazid and rifampin who are treatment intolerant or nonresponsive to standard therapy, based on a multicenter phase 3 trial, Nix-TB, that enrolled 109 patients. The external controls included a literature review of outcomes in patients treated for extensively drug-resistant tuberculosis excluding the 3 test drugs and a matched historical control group of patients treated for extensively drug-resistant tuberculosis at one of the Nix-TB study centers (www.tballiance.org/downloads/NixTB/NixTB_factsheet.pdf). The matched historical control group partially addressed the limitations of the literature review related to heterogeneity, publication bias, and lack of comparability in terms of geography, patient characteristics, and study assessments.

Among the initiatives that would substantially help in establishing databases for rare IFDs are clearly specified guidance that would support regulatory approval of investigational agents. As design, development, and operation of such databases requires resources, funding from government agencies, industry, and foundations to collaborative groups, including but not limited to the Mycoses Study Group Education & Research Consortium, Fungal Diagnostic Laboratory Consortium, the European Confederation for Medical Mycology, the International Society for Human and Animal Mycology, and the European Society for Clinical Microbiology and Infectious Diseases Fungal Infection Study Group, is vital to create these important data infrastructures.

## ANIMAL MODELS

Carefully designed studies of investigational antifungal agents in predictive laboratory animal models of rare IFDs could provide important data to inform an understanding of the activity of the novel drug as compared to the approved or standard of care drug(s). The nonclinical studies of isavuconazonium in treatment of experimental pulmonary and disseminated mucormycosis exemplify this approach. Luo et al [71]demonstrated that isavuconazonium (215 mg/kg thrice daily by oral gavage) significantly prolonged survival and reduced the tissue fungal burden in immunocompromised mice with experimental pulmonary mucormycosis produced by *R. delemar*. The activity of isavuconazonium at this dosage was comparable to that of liposomal amphotericin B (intravenous; 15 mg/kg/d) in prolonging survival and reducing the tissue fungal burden.

These in vivo observations were consistent with, and supportive of, the findings in the single-arm open-label clinical trial (VITAL study) reported by Marty et al [30], in which the crude all-cause mortality rate on day 42 was 33% (7 of 21) in case patients treated with isavuconazonium as primary therapy, similar to that in amphotericin B–treated matched controls (39% [13 of 33]), with weighted all-cause mortality rates of 33% versus 41% (*P* = .595). In developing new antifungal agents for treatment of rare IFDs, animal model systems can be used for studying mucormycosis [72–74], scedosporiosis [75–79], fusariosis [79, 80], trichosporonosis [81–83], and candidiasis caused by *C auris* [84–86]. These model systems may be useful for understanding the activity of investigational antifungal agents against rare IFDs.

## EFFICACY END POINTS

Mortality and global clinical response criteria are used as clinical end points in clinical trials of IPA and could be used in studies of rare IFDs. For assessment of efficacy of therapies in IFDs, mortality rates may be analyzed as all-cause mortality rate or fungal-free survival rate. A mortality end point has some advantages in an open-label historically controlled study due to its objective measurement, standardized reporting across studies, and clinical importance. Disadvantages of using all-cause mortality would include the sample size required and the cofounding effect of comorbid conditions; however, a large difference in treatment effect of the test drug over the control group could be significant despite low numbers of patients in the trial. Fungal-free survival also has limitations as an end point, especially in determining the presence or absence of active infection. Global clinical response, which has been used as a reliable end point in clinical trials for candidemia, may also be valid in studies of rare IFDs [87, 88].

A successful demonstration of efficacy may be defined as showing superiority to existing therapy or, when appropriate, showing noninferiority to standard of care treatment. While superiority of a new antifungal agent over existing therapy would be an ideal outcome, an alternative outcome of noninferiority with an antifungal agent with other advantages, such as reduced toxicity, increased tolerability, and more favorable pharmacokinetics, could also be considered a therapeutic advance. A desirability of outcome ranking analysis would allow incorporation of toxicity in a noninferiority outcome analysis [89]. In a noninferiority trial there should be evidence from previous studies that the standard of care used in the control group is highly effective. One could also consider the role for an independent review committee to adjudicate the outcomes of patients in a clinical trial in a manner similar to that used for some oncologic therapeutics and the antibacterial drug daptomycin [90].

As none of the currently available diagnostic tests have sufficient sensitivity and specificity when used alone, the optimal approach to assessment of efficacy relies on a combination of multiple diagnostic strategies. The treatment response to antifungal therapy may be monitored using innovative use of biomarkers, such as changes from baseline in serum galactomannan, quantitative polymerase chain reaction signals for detection of fungal DNA in whole blood, and imaging techniques, such as high-resolution computed tomography and positron emission tomography to detect changes in lesion size or resolution [91]. Time points for outcome assessment should be similar between patients in the study and historical controls. Patient-reported outcome measures may be helpful as end points in chronic mycoses; however, there are no widely available patient-reported outcome instruments for the study of rare IFDs.

## ASSESSMENT OF SAFETY

Patient-level safety data at the intended dose and duration of treatment of the investigational drug should be evaluated. Safety data from human pharmacokinetic/safety studies, published clinical studies of the study drug's use in the treatment of other diseases, patient registry database(s), case series, and case reports could also be supportive. Discussions with regulatory agencies early in the development program are important to reach consensus on the types of clinical and nonclinical studies to be conducted, as well as the size of the safety database. For those drugs already approved for more common IFDs, the size of the existing safety database in patients may be sufficiently informative. For new molecular entities, discussion is warranted to determine the adequacy of the safety database.

While the approval of isavuconazole for primary treatment of mucormycosis was based on the efficacy data of the VITAL study [30], the safety data supporting that decision were obtained from the SECURE study [1]. Thus, a balance is needed between potentially sparse data sets for efficacy of an antifungal agent for rare IFDs and safety, where a larger sample size is needed. While the safety of medications used for treatment of rare cancers and inborn errors of metabolism could be assessed within the population intended for use, antifungal agents will likely be used for treatment of a wider range of patients with IFDs, for which approval has not been granted.

## ALTERNATIVE APPROACHES TO MEETING CHALLENGES OF RARE IFDs

Repurposing drugs that were not originally developed as antifungal agents may be a pragmatic solution for treatment of rare IFDs [92, 93]. A wide range of drugs that are licensed for other purposes have been found to have in vitro and in vivo antifungal activity against common fungal pathogens [92]. Zhang and colleagues [93] discuss a wide range of licensed antibacterial agents, immunomodulatory drugs, statins, and nonsteroidal anti-inflammatory drugs that exert in vitro and in vivo activity, alone or in combination with antifungal agents against common fungal pathogens.

As a case in point, miltefosine, an orally administered hexadecyclphosphocholine used in the treatment of visceral, cutaneous, and mucosal leishmaniasis, has been advocated for repurposed use for treatment of uncommon but emerging causes of IFDs, including *C auris*, the Mucorales, *Scedosporium* spp, and *Lomentospora* spp [94–96]. Consistent with this repurposing, the FDA has approved the compassionate use/expanded access to miltefosine for refractory *Scedosporium* infections. More studies focusing on those agents with known favorable safety profiles are needed to characterize their activity and potential utility against the less common fungal pathogens.

When randomized trials are not feasible for assessing a rare disease, observational data may be used to emulate a target trial [97–99]. Using appropriate methodology, the process of target trial emulation applies the study design concepts of a randomized trial to observational data in order to estimate the causal effect of an intervention [97]. For example, target trial emulation methods were used in the COHERE study, which evaluated the potential benefit of withholding primary prophylaxis against *Pneumocystis* pneumonia in patients with HIV [98]. Using a target trial emulation approach, this study demonstrated that primary prophylaxis against *Pneumocystis* pneumonia in patients with HIV could be safely withdrawn in virologically suppressed patients on antiretroviral therapy, independent of their CD4 cell count [98]. As another example of using target emulation methods, marginal structural models were developed to emulate a randomized clinical trial from several clinical cohort databases, which found no significant difference in all-cause mortality rates, when comparing the impact of antiretroviral therapy started early (≤14 days after diagnosis of cryptococcal meningitis) versus late (14–56 days after diagnosis) in HIV-infected patients from high-income countries [99].

## PATIENT ADVOCACY

Paramount to the mission of developing new antifungal agents is to relieve suffering and improve outcomes patients with IFDs. As patients with cancer or inborn errors of metabolism may have strong advocacy groups in support of new therapeutic options, there has historically been a paucity of such patient advocacy for IFDs. Programs of the Valley Fever Institute for coccidioidomycosis and the Henry Schueler Foundation for mucormycosis are notable exceptions to this dearth of mycologic advocacy. However, through the collaborative efforts of patients, caregivers, physicians, and advocacy experts, there is hope for patients’ voices to be heard in the need for new antifungal agents and diagnostics through a recently established organization known as Mycology Advocacy, Research & Education (MyCARE) [100].

Among the important priorities in development of new antifungal agents is the licensed indication for their IFDs. Patients have expressed grave concern about third-party payers denying support for the use of an antifungal agent for a nonlicensed but clinically indicated use against an uncommon IFD. Regulatory approval of an antifungal agent for rare IFDs provides tenable support for reimbursement and a relief of fiscal distress for a patient who is already experiencing the damage caused by the infection.

## FUTURE DIRECTIONS

To advance pathways for antifungal drug development for rare IFDs, and to build on the 2020 FDA workshop on antifungal drugs [101, 102], continued engagement with stakeholders is essential to further develop a consensus among clinical investigators, industry, the FDA, and other regulatory agencies, such as the European Medicines Agency. Given that there are emerging novel antifungal agents with activity against rare fungal pathogens, a timely workshop to discuss innovative trial designs would help to advance the field. In addition to panelists with infectious diseases expertise, participation of members of the Division of Rare Diseases and Medical Genetics and the Office of Oncologic Diseases, as well as representatives from the Rare Disease Drug Development Council at the FDA and other regulatory agencies, may provide valuable perspective and experience.
